# Large Community Outbreak of Legionnaires Disease Potentially Associated with a Cooling Tower — Napa County, California, 2022

**DOI:** 10.15585/mmwr.mm7249a1

**Published:** 2023-12-08

**Authors:** Nárjara V. Grossmann, Crystal Milne, Melinda R. Martinez, Karen Relucio, Banafsheh Sadeghi, Erica N. Wiley, Samuel N. Holland, Sarah Rutschmann, Duc J. Vugia, Akiko Kimura, Chad Crain, Farhima Akter, Rituparna Mukhopadhyay, John Crandall, Meghann Shorrock, Jessica C. Smith, Namrata Prasad, Rebecca Kahn, Albert E. Barskey, Sooji Lee, Melisa J. Willby, Natalia A. Kozak-Muiznieks, Claressa E. Lucas, Kelley C. Henderson, Jennafer A. P. Hamlin, Eungi Yang, Nakia S. Clemmons, Troy Ritter, Jennifer Henn

**Affiliations:** ^1^Public Health Division, Napa County Health & Human Services Agency, Napa, California; ^2^Infectious Diseases Branch, California Department of Public Health; ^3^Drinking Water and Radiation Laboratory, California Department of Public Health; ^4^Microbial Diseases Laboratory, California Department of Public Health; ^5^Division of Bacterial Diseases, National Center for Immunization and Respiratory Diseases, CDC; ^6^Epidemic Intelligence Service, CDC; ^7^ASRT, Inc., Atlanta, Georgia; ^8^Division of Environmental Health Science and Practice, National Center for Environmental Health, CDC.

**Summary**

**What is already known about this topic?**

Legionnaires disease is a serious pneumonia caused by *Legionella* bacteria. Molecular analysis that compares clinical and environmental *L.* *pneumophila* isolates allows for identification of associations among possible sources of disease.

**What is added by this report?**

In a large Legionnaires disease outbreak in California in July 2022, sequence-based typing, in tandem with nucleotide polymorphism analysis linked one *Legionella* sequence type to a cooling tower and two cases. Mapping facilitated targeted sampling and remediation.

**What are the implications for public health practice?**

Timely source identification and remediation effectively halt disease spread. Prompt collection of respiratory specimens, paired with targeted environmental sampling, facilitates comparison with environmental samples for source attribution; culture-independent typing methods are useful when isolates are not recovered from clinical specimens.

## Abstract

Legionnaires disease is a serious infection acquired by inhalation of water droplets from human-made building water systems that contain *Legionella* bacteria. On July 11 and 12, 2022, Napa County Public Health (NCPH) in California received reports of three positive urinary antigen tests for *Legionella pneumophila* serogroup 1 in the town of Napa. By July 21, six Legionnaires disease cases had been confirmed among Napa County residents, compared with a baseline of one or two cases per year. NCPH requested assistance from the California Department of Public Health (CDPH) and CDC to aid in the investigations. Close temporal and geospatial clustering permitted a focused environmental sampling strategy of high-risk facilities which, coupled with whole genome sequencing results from samples and investigation of water system maintenance, facilitated potential linking of the outbreak with an environmental source. NCPH, with technical support from CDC and CDPH, instructed and monitored remediation practices for all environmental locations that tested positive for *Legionella.* The investigation response to this community outbreak illustrates the importance of interdisciplinary collaboration by public health agencies, laboratory support, timely communication with the public, and cooperation of managers of potentially implicated water systems. Timely identification of possible sources, sampling, and remediation of any facility testing positive for *Legionella* is crucial to interrupting further transmission.

## Investigation and Results

### Epidemiologic Investigation

Napa County Public Health (NCPH) defined a confirmed case as the diagnosis of Legionnaires disease based on the results of a urinary antigen test (UAT), polymerase chain reaction (PCR) test, or culture received by a person who lived, worked, or spent time in downtown Napa, with illness onset during or after June 2022. A suspected case was defined as community-acquired pneumonia of unknown origin identified among three categories of persons: 1) a hospitalized patient; 2) a resident of, worker in, or visitor to downtown Napa; or 3) a patient who did not receive testing for *Legionella* spp. during hospitalization.

During July 11–August 15, 2022, NCPH identified 17 Legionnaires disease cases, including 14 confirmed and three suspected cases (Table 1). Among these 17 cases, 16 persons were hospitalized, 10 were admitted to an intensive care unit, and five required intubation and mechanical ventilation; one patient died. Comorbidities included smoking, diabetes, hypertension, lung disease, and heart disease. Two patients were coinfected with SARS-CoV-2, the virus that causes COVID-19. The longest hospital stay was 36 days. All confirmed cases were diagnosed by UAT results. Lower respiratory tract specimens were collected from four patients with confirmed Legionnaires disease; *L. pneumophila* serogroup 1 was detected by PCR in two clinical specimens, one of which yielded an isolate, which is necessary for whole genome sequencing. Interviews with patients or their proxies revealed that 14 patients lived in downtown Napa, two visited downtown Napa, and one worked in downtown Napa.

**TABLE 1:** Selected characteristics of patients with confirmed* and suspected^†^ Legionnaires disease — Napa County, California, 2022

Characteristic	Legionnaires disease cases, no. (%)	
	Confirmed, n = 14*	Suspected, n = 3^†^
Age, yrs, mean (range)	62.6 (47–83)	62.7 (—^§^)
Age, yrs, median	64	—^§^
Male sex	12 (86)	1 (33)
**Hospitalized (% of total cases)**	13 (93)	3 (100)
ICU admission	10 (71)	0 (—)
Intubated	5 (36)	0 (—)
**Residence zone**		
High-risk^¶^	11 (79)	3 (100)
Low-risk**	3 (21)	0 (—)
Total living in or with visits to high-risk zone	14 (100)	3 (100)
**Hospital length of stay, days, mean (range)**	10.4 (2–36)	5.7 (3–9)
**Days from onset to diagnosis, days, mean (range)**	8.8 (4–13)	NA
**Comorbidities**		
Coronary heart disease	5 (36)	0 (—)
SARS-CoV-2 coinfection	2 (14)	0 (—)
Current or former smoker	11 (79)	2 (67)
Diabetes	4 (29)	0 (—)
Hypertension	5 (36)	1 (33)
Lung disease	6 (43)	0 (—)

**Abbreviations:** ICU = intensive care unit; NA = not applicable.

* Legionnaires disease was confirmed among 14 patients based on the results of a urinary antigen test, polymerase chain reaction test, or culture received by those who lived, worked, or spent time in downtown Napa, with illness onset during or after June 2022.

^†^ Community-acquired pneumonia of unknown origin was identified among three categories of persons: 1) a hospitalized patient; 2) a resident of, worker in, or visitor to downtown Napa; or 3) a patient who did not receive testing for *Legionella* spp. during hospitalization.

^§^ Data suppressed for patient privacy.

^¶^ The area within a 1.0-mile (1.6-km) radius from the center of a circle surrounding the patients’ residences plotted on a point density heat map using ArcGIS Pro (version 3.0; Esri).

** Areas not within the high-risk zone.

### Environmental Health Investigation

The search for potential environmental sources began with the delimitation of a high-risk zone, which was defined as the area within a 1.0-mile (1.6-km) radius from the center of a circle drawn around the cluster of patients’ residences plotted on a point density heat map generated using ArcGIS Pro (version 3.0; Esri) (Figure). Aerial imagery, onsite visual inspections, and calls to businesses and cooling tower maintenance companies identified and confirmed the locations and uses of cooling towers.* Environmental sampling locations were selected on the basis of patient interviews, and a risk score analysis was derived from the geographic proximity of facilities with cooling towers and other aerosolizing devices to the patients’ residences. A total of seven facilities with nine potential exposure sources (seven cooling towers, one decorative fountain, and one produce mister) were mapped within the high-risk zone (Figure) (Table 2). Cooling towers located at facilities A and B were the highest scoring devices in the risk score analysis.

**FIGURE:**
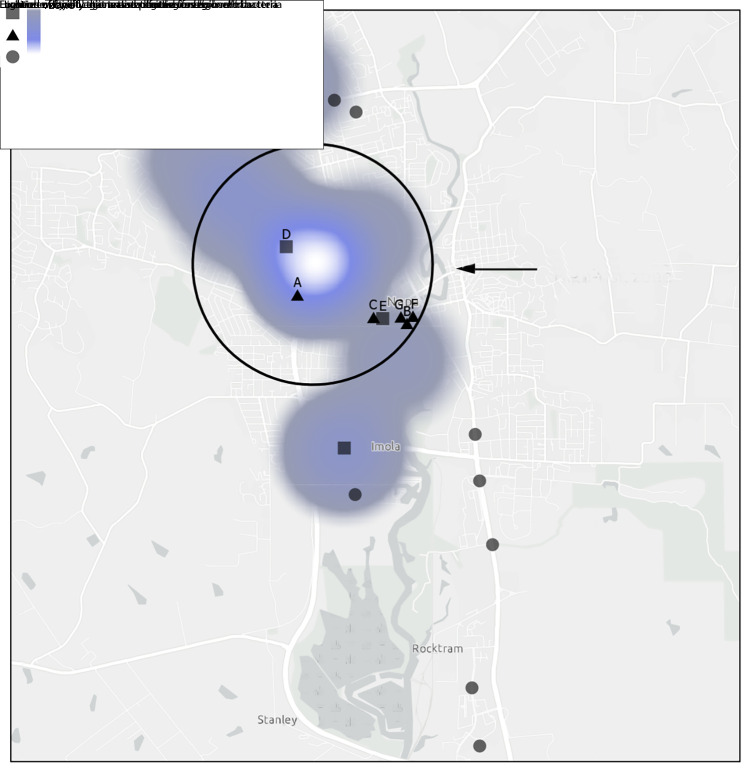
Point density heat map of residences of patients with Legionnaires disease — Napa County, California, 2022* **Sources:** County of Napa; California State Parks; Esri; HERE Technologies; Garmin International; SafeGraph; GeoTechnologies, Inc.; Ministry of Economy, Trade, and Industry of Japan/National Aeronautics and Space Administration; United States Geological Survey; Bureau of Land Management; Environmental Protection Agency; National Park Service; United States Department of Agriculture. * The high-risk zone is defined as the area within a 1.0-mile (1.6-km) radius from the center of a circle surrounding the patients’ residences plotted on a point density heat map generated using ArcGIS Pro (version 3.0; Esri).

**TABLE 2:** Potential *Legionella* sources within and outside the high-risk zone* with respect to the type of device, culture or polymerase chain reaction test results, and sequence types identified — Napa County, California, 2022

Facility	Type of device	Within high-risk zone	Sampled by public health authorities	Detection by culture or PCR	Sequence-based typing result
A	Cooling tower	Yes	Yes	*L. pneumophila*	ST35
A	Decorative fountain	Yes	Yes	*L. pneumophila*	ST1
B	Cooling tower	Yes	Yes	*L. pneumophila*	ST1
C	Cooling tower	Yes	Yes	*L. pneumophila* and *L. anisa*^Ϯ^	NA
D	Produce mister	Yes	Yes	No *Legionella* detected	NA
E	Cooling tower	Yes	Yes	No *Legionella* detected	NA
F	Cooling tower 1	Yes	Yes	*L. pneumophila*	ST296
F	Cooling tower 2	Yes	Yes	*L. pneumophila*	ST296
G	Cooling tower	Yes	Yes	*L. pneumophila*	ST10
H	Hot tub	No	Yes	No *Legionella* detected	NA
H	Decorative fountain	No	Yes	No *Legionella* detected	NA
I	Multiple cooling towers	No	No	No *Legionella* detected^§^	NA
J	Cooling tower	No	No	NA	NA
K	Cooling tower	No	No	NA	NA
L	Multiple cooling towers	No	No	No *Legionella* detected^§^	NA
M–S	Cooling towers	No	No	NA	NA

**Abbreviations**: NA = not applicable; PCR = polymerase chain reaction; ST = sequence type.

* The area within a 1.0-mile (1.6-km) radius from the center of a circle surrounding the patients’ residences plotted on a point density heat map generated using ArcGIS Pro (version 3.0; Esri).

^Ϯ^ CDC’s Pneumonia Response and Surveillance Laboratory detected *Legionella anisa*, whereas testing at an independent private laboratory arranged by the facility shortly after sampling by public health authorities detected *L. pneumophila*.

^§^ Self-tested: facility voluntarily collected environmental samples and arranged testing for *Legionella* at a commercial laboratory.

Visual inspection, review of records, and sampling of devices within the high-risk zone revealed a lack of maintenance at most cooling towers. Many had low or no detectable chlorine at the time of sampling, because of lack of routine biocide application, improper distribution methods, or other problems with the system.^†^ Facility A’s cooling tower had a clog in the pipe leading to the chemical feed system that impeded the controller’s ability to detect water flow, resulting in low or no injection of biocide into the tower. According to maintenance records, the clog was detected in early July, at approximately the same time that many case exposures occurred and was resolved in early August.

Public health investigators collected environmental samples from 11 potential sources. Seven samples tested positive for *Legionella* (six for *L. pneumophila* only and one for both *L. pneumophila* and *Legionella anisa*); all positive samples were collected within the high-risk zone.

### Laboratory Investigation

*L. pneumophila* culture–positive clinical and environmental specimens underwent sequence-based typing at CDC and whole genome sequencing followed by single-nucleotide polymorphism (SNP) analysis at the California Department of Public Health (CDPH). Sequence-based typing generates an allelic profile based on the combination of allele numbers at seven loci (*1*). Each unique allelic profile corresponds to a sequence type (ST). Nested sequence-based typing, a culture-independent variation of sequence-based typing, was performed on the PCR-positive clinical specimen from which no isolate was recovered. In SNP analysis, whole genome sequencing data generated from isolates are aligned to a reference genome, and the variation from the reference is used to infer relatedness among isolates, visualized in a phylogenetic tree. A smaller number of SNP differences indicates closer relatedness (*2*).

The identified *L. pneumophila* STs from environmental samples included ST1, ST10, ST35, and ST296 (Table 2). ST35 was detected in the clinical isolate via sequence-based typing. Nested sequence-based typing performed on the PCR-positive, culture-negative clinical specimen also detected ST35. The only environmental sample that yielded ST35 was collected from the facility A cooling tower. No SNP differences between the clinical isolate and the facility A cooling tower isolate were identified, indicating that they were highly related, whereas other environmental isolates were genetically distant from facility A’s cooling tower isolate (Supplementary Figure, https://stacks.cdc.gov/view/cdc/136165). This activity was reviewed by CDC, deemed not research, and was conducted consistent with applicable federal law and CDC policy.^§^

## Public Health Response

A coordinated public communication strategy was implemented. An outbreak alert was sent to local health care providers, requesting *Legionella* testing for hospitalized patients with community-acquired pneumonia or those failing outpatient treatments. CDPH notified other local health departments, and a public press release encouraged persons with symptoms consistent with Legionnaires disease to seek care. A public-facing webpage with information about the outbreak was created on the Napa County website.^¶^

The heat map and high-risk zone definition served as the basis for prioritizing environmental testing resources to devices most likely to have generated aerosols to which patients in this cluster were exposed. Facilities where *Legionella* was detected were notified to immediately begin remediation of their cooling towers.** One facility that did not respond to oral and written communications received a legal order to shut down its cooling tower until remediation was completed. NCPH tracked remediation efforts and, when available, inspected remediation logs and maintenance records. The last Legionnaires disease case was detected on August 15, by which time most facilities had initiated or completed remediation. Facilities with cooling towers outside the high-risk zone were informed of the outbreak and best practices for cooling tower maintenance.

## Discussion

Similarities between symptoms of COVID-19 and Legionnaires disease pose challenges to investigating community clusters of Legionnaires disease, including a risk for delayed care, resulting in worse outcomes if symptoms are presumed to be caused by COVID-19. In this investigation, patient interviews and risk score analysis narrowed the environmental investigation to a few devices in downtown Napa as potential sources of the outbreak. The period between identification of the clog that impeded adequate biocide delivery at facility A’s cooling tower and its remediation approximately coincided with the onset of Legionnaires disease cases. Identification of ST35 in two patient specimens and identical SNP results between the clinical and cooling tower isolates further support a potential causal link between facility A and the outbreak. This report is the first to identify ST35 in a California Legionnaires disease outbreak; previous ST35 outbreaks were identified in Mississippi, Nevada, and the U.S. Virgin Islands. ST35 strains might possess enhanced ability to cause disease and might be resistant to standard remediation efforts, resulting in reappearance after disinfection (*3*).

Despite robust surveillance, no cases were detected among occupants of facility A. Studies show that cooling towers can spread *Legionella* over a wide geographic area, with highest attack rates among persons living within 0.6 miles (1.0 km) of the tower (*4*,*5*). This investigation further highlights the risks cooling towers can pose for susceptible persons in surrounding neighborhoods. Cooling towers without a comprehensive water management program or lacking routine maintenance are associated with an increased risk for *Legionella* colonization (*6*,*7*). Even after an outbreak, building owners and managers might not always follow best water management practices (*8*). A close relationship between public health sectors and local businesses, along with guidance on recommended operation and maintenance of water systems, can help prevent further outbreaks.

### Public Health Practice

A coordinated public health response was critical to the investigation of and response to this outbreak. Support from CDC and state health departments during Legionnaires disease outbreak investigations provide *Legionella*-specific subject matter expertise and laboratory capacity for environmental testing for local health jurisdictions lacking these resources. Furthermore, restricting the search area and maintaining active communication with local businesses facilitate investigation and response activities. Finally, molecular analyses of clinical specimens and environmental samples, including culture-independent techniques such as nested sequence-based typing, are powerful resources in the investigation of Legionnaires disease outbreaks. Timely identification of possible sources, sampling, and remediation of any facility testing positive for *Legionella* are crucial to interrupting further transmission. Facilities should comply with best practices for cooling tower maintenance such as having a water management program that includes routine maintenance and water quality parameters surveillance (*7*).

## Footnotes

* A cooling tower is a centralized heat-rejection system for buildings or industrial processes that uses water and fans to remove heat from the air.

† A typical cooling tower has an automated chemical feed system to inject water treatment chemicals. This system has two primary components: a pump that injects the chemicals (including biocides) and a controller that tells the system when and at what speed and volume to inject the chemicals.

§ 45 C.F.R. part 46 102(1)(2), 21 C.F.R. part 56; 42 U.S.C. Sect. 241(d); 5 U.S.C. Sect. 552a; 44 U.S.C. Sect. 3501 et seq.

¶
https://www.countyofnapa.org/3370/Legionnaires-Disease

** Cleaning and disinfection procedures for cooling towers associated with an outbreak are available in the cooling tower module of the CDC *Legionella* Control Toolkit. https://www.cdc.gov/legionella/wmp/control-toolkit/cooling-towers.html

## References

[1] Lück C, Fry NK, Helbig JH, Jarraud S, Harrison TG. Typing methods for *Legionella*. In: Buchrieser C, Hilbi H, eds. Methods in molecular biology: *Legionella*. Totowa, NJ: Humana Press; 2013. https://link.springer.com/protocol/10.1007/978-1-62703-161-5_6 10.1007/978-1-62703-161-5_6 23150392

[2] Kozyreva VK, Truong CL, Greninger AL, Crandall J, Mukhopadhyay R, Chaturvedi V. Validation and implementation of Clinical Laboratory Improvements Act–compliant whole-genome sequencing in the public health microbiology laboratory. J Clin Microbiol 2017;55:2502–20. 10.1128/JCM.00361-17 28592550 PMC5527429

[3] Kozak-Muiznieks NA, Lucas CE, Brown E, Prevalence of sequence types among clinical and environmental isolates of *Legionella pneumophila* serogroup 1 in the United States from 1982 to 2012. J Clin Microbiol 2014;52:201–11. 10.1128/JCM.01973-13 24197883 PMC3911437

[4] Addiss DG, Davis JP, LaVenture M, Wand PJ, Hutchinson MA, McKinney RM. Community-acquired Legionnaires’ disease associated with a cooling tower: evidence for longer-distance transport of *Legionella pneumophila.* Am J Epidemiol 1989;130:557–68. 10.1093/oxfordjournals.aje.a115370 2764000

[5] Bhopal RS, Fallon RJ, Buist EC, Black RJ, Urquhart JD. Proximity of the home to a cooling tower and risk of non-outbreak Legionnaires’ disease. BMJ 1991;302:378–83. 10.1136/bmj.302.6773.378 2004142 PMC1676166

[6] Mouchtouri VA, Goutziana G, Kremastinou J, Hadjichristodoulou C. *Legionella* species colonization in cooling towers: risk factors and assessment of control measures. Am J Infect Control 2010;38:50–5. 10.1016/j.ajic.2009.04.285 19699013

[7] Garrison LE, Kunz JM, Cooley LA, Vital signs: deficiencies in environmental control identified in outbreaks of Legionnaires’ disease—North America, 2000–2014. MMWR Morb Mortal Wkly Rep 2016;65:576–84. 10.15585/mmwr.mm6522e1 27281485

[8] Bhopal RS, Barr G. Maintenance of cooling towers following two outbreaks of Legionnaires’ disease in a city. Epidemiol Infect 1990;104:29–38. 10.1017/S0950268800054492 2307183 PMC2271738

